# A comparison of the isometric force fatigue-recovery profile in two posterior chain lower limb tests following simulated soccer competition

**DOI:** 10.1371/journal.pone.0206561

**Published:** 2019-05-03

**Authors:** Anton Matinlauri, Pedro E. Alcaraz, Tomás T. Freitas, Jurdan Mendiguchia, Afshin Abedin-Maghanaki, Alberto Castillo, Enrique Martínez-Ruiz, Jorge Carlos-Vivas, Daniel D. Cohen

**Affiliations:** 1 UCAM Research Center for High Performance Sport, Catholic University of Murcia, Murcia, Spain; 2 Department of Performance, HJK Helsinki, Helsinki, Finland; 3 Faculty of Sport Sciences, UCAM, Catholic University of Murcia, Murcia, Spain; 4 Department of Physical Therapy, Zentrum Rehabilitation and Performance Center, Pamplona, Spain; 5 Department of Physical Therapy, EMR Rehab and Prevention Center, Elche, Spain; 6 Faculty of Health Sciences, University of Santander (UDES), Bucaramanga, Colombia; University of Belgrade, SERBIA

## Abstract

**Aim:**

To evaluate the reliability of isometric peak force (IPF) in a novel “long-length” 90°Hip:20°Knee (90:20) strength test and to compare the simulated soccer match induced fatigue-recovery profile of IPF in this test with that of an isometric 90°Hip:90°Knee (90:90) position test.

**Methods:**

Twenty semi-professional soccer players volunteered for the study of which 14 participated in the first part of the study which assessed 90:20 reliability (age = 21.3 ± 2.5 years, height = 1.79 ± 0.07 m, body mass = 73.2 ± 8.8 kg), while 17 completed the second part of the study evaluating fatigue-recovery (age 21.2±2.4 yrs., height = 180 ± 0.09 m, body mass 73.8 ± 8.9 kg). We evaluated the inter-session reliability of IPF in two 90:20 test protocols (hands on the wall (HW); and hands on chest (HC)) both performed on two occasions, 7 days apart. We then assessed 90:20 (HC) and 90:90 IPF immediately before (PRE) and after (POST) after a simulated soccer match protocol (BEAST90mod) and 48 (+48 h) and 72 hours (+72 h) later.

**Results:**

Part one: the 90:20 showed moderate to high overall reliability (CV’s of 7.3% to 11.0%) across test positions and limbs. CV’s were lower in the HW than HC in the dominant (7.3% vs 11.0%) but the opposite happened in the non-dominant limb where CV’s were higher in the HW than HC (9.7% vs 7.3%). Based on these results, the HC position was used in part two of the study. Part two: 90:20 and 90:90 IPF was significantly lower POST compared to PRE BEAST90mod across all testing positions (p<0.001). IPF was significantly lower at +48 h compared to PRE in the 90:20 in both limbs (Dominant: p<0.01,Non-dominant: p≤0.05), but not in the 90:90. At +72 h, IPF was not significantly different from PRE in either test.

**Conclusions:**

Simple to implement posterior IPF tests can help to define recovery from competition and training load in football and, potentially, in other multiple sprint athletes. Testing posterior chain IPF in a more knee extended 90:20 position may provide greater sensitivity to fatigue at 48 h post simulated competition than testing in the 90:90 position, but also may require greater degree of familiarization due to more functional testing position.

## Introduction

Hamstring strain injuries (HSI) are the most common injury in multiple sprint team sports such as soccer [1] and a professional team of 25 players can expect approximately 5–7 hamstring strains per season [1,2] which can have a significant impact on team performance and success [3]. It is well recognized that HSI risk is determined by the interaction between a number of non-modifiable and modifiable risk factors [4]. Amongst modifiable risk factors, there is particular interest in neuromuscular performance characteristics that can be screened for, and potentially addressed in training interventions, such as inadequate hamstrings strength [5] and inter-limb strength imbalances [6,7].

Significant decreases in isokinetic and isometric hamstrings strength are reported immediately after simulated and real competition [8,9] and in the subsequent 24–72 hours and beyond [10,11]. Given that acute fatigue within matches [12] and accumulated fatigue—represented by fixture congestion [13]—are implicated as risk factors for HSI, it is speculated that decrements in strength [8,9], and/or increases in strength asymmetry [14] may be a contributory factor to the fatigue-injury association observed in epidemiological studies [12]. Therefore, one approach that may reduce HSI risk is to regularly assess player’s hamstrings strength in-season between competitive matches [14–17] in order to identify significant changes which may represent excessive muscle fatigue/inadequate recovery or adaptation, information which may then inform decisions on training load, match participation and / or recovery strategies, or warrant further examination of the athlete.

As frequent muscle strength assessment using “gold standard” isokinetic dynamometry is time-consuming and impractical in team sport environments, alternative means to rapidly evaluate hamstrings/posterior chain muscle strength and changes in strength asymmetry in-season have been proposed. Schache et al [14] described a 3 sec isometric supine posterior lower limb muscle test performed in two supine positions; 90°:90° (knee:hip flexion) (90°:90°) and 30° (knee flexion) and McCall et al [15] and Nedelec et al [10] subsequently demonstrated the sensitivity of both positions to detect acute [15] and residual [10] fatigue following soccer match play. Using a fixed dynamometer hamstrings test performed in a position representing the terminal swing phase of running, Wollin et al [16] observed significant decreases in IPF immediately post and 24 hours following competition in elite youth soccer with similar findings reported in Australian Rules football [17]. Evidence suggests that 83% of HSI’s occur to the Biceps Femoris (BF) [18] during terminal swing, when a combination of factors cause the BF to be biomechanically exposed. In this phase, when the hip is highly flexed (55–65°), and the knee is slightly flexed (30–45°) the BF is at its peak length [18,19], and as it has a larger hip extension than knee flexion moment arm, hip flexion produces greater relative lengthening of the BF than the other hamstring muscles [19]. Furthermore, Cohen et al [8] found that immediately following simulated soccer competition, the largest decrement in isokinetic eccentric hamstring peak torque was observed in the most lengthened position tested– 90° of hip flexion and 10° of knee extension. It is therefore of interest to determine whether match induced acute and residual strength decrements are also greater when measured with the hamstrings in a more extended position in an isometric test which can feasibly be implemented regularly in a team sports setting. Given the limited time available for testing in this setting in-season, performing a smaller number of tests—potentially supporting the implementation of a bilateral test capturing the mean response of both limbs. However, evidence suggests that the fatigue response differs in the dominant and non-dominant limbs, with larger match-induced acute and residual deficits in isometric posterior chain force reported in the dominant limb. However, the association between fatigue in the two limbs has not been examined making the importance of testing both limbs difficult to assess.

Therefore, the principle aims of the present study were: [1] to evaluate the reliability of a novel standing isometric posterior chain test (90°_Hip_:20°_Knee_) force platform in professional footballers; and [2] to compare the fatigue-recovery profile of this test with the supine 90°:90° test described by McCall et al [15]. In addition, we aimed to evaluate the correlation between the dominant and non-dominant limb fatigue.

We hypothesised that the simulated match would lead to larger decreases in 90:20 than 90:90 IPF.

## Methods

### Subjects

Twenty semi-professional male football players from a Spanish third division club volunteered for the study. Fourteen participants (age = 21.3 ± 2.5 years, height = 1.79 ± 0.07 m, body mass = 73.2 ± 8.8 kg) were included and able to complete all testing procedures in the first part of the study; 90:20 reliability. Seventeen participants (age = 21.2 ± 2.4 yrs., height = 180 ± 0.09 m, body mass 73.8 ± 8.9 kg) were included and able to complete all testing protocols required in the second part; fatigue-recovery response.

Players were excluded if (i) they had pre-existing conditions or injuries that could affect test performance; (ii) experienced lower limb muscle soreness or pain before or during testing; and (iii) participated in moderate or high intensity training involving the lower limbs in the 3 days prior to the testing day.

**Fig 1:**
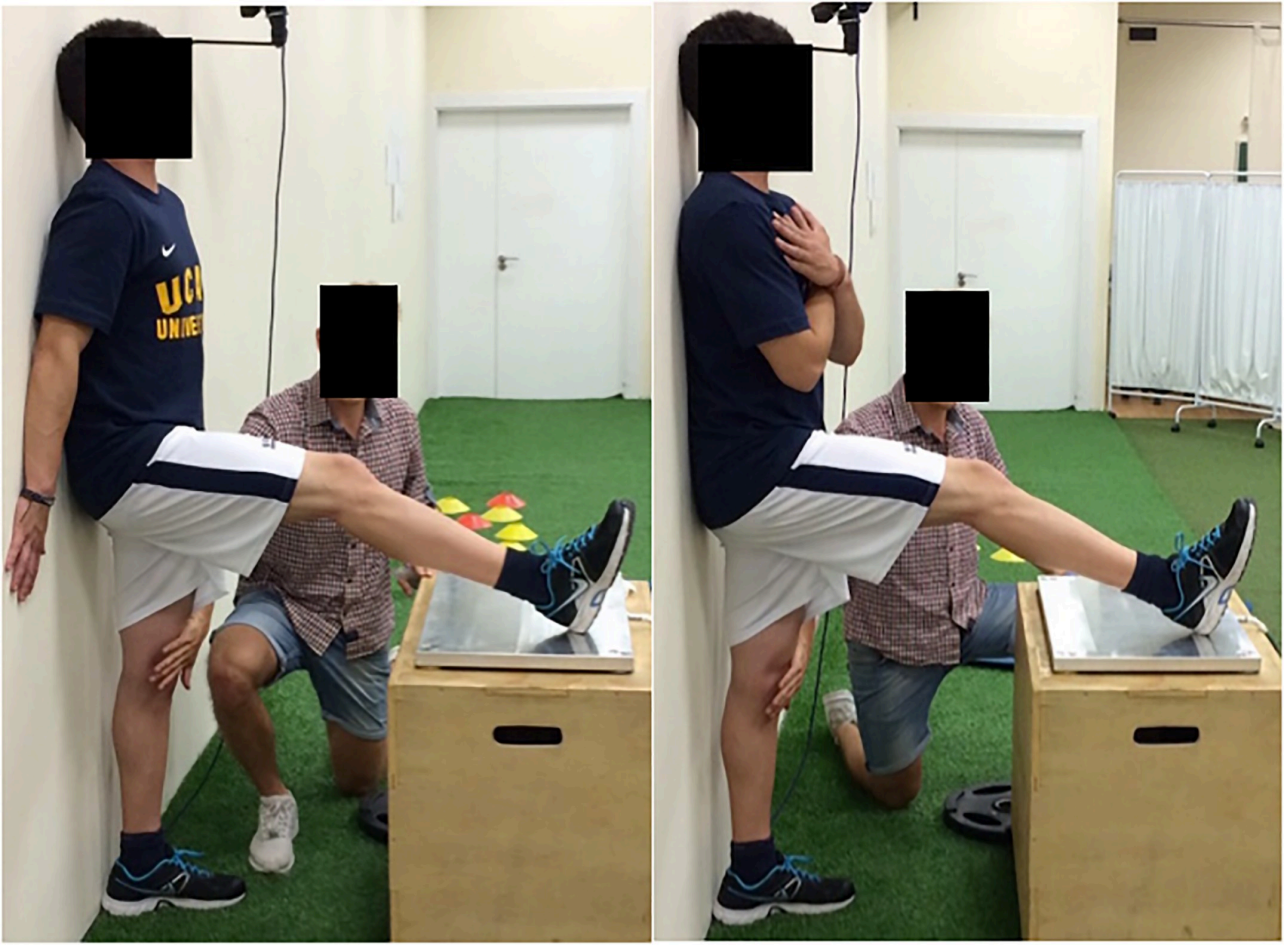
90°:20° protocols. (a) with hands crossed on chest (HC) (b) with hands against the wall (HW).

**Fig 2:**
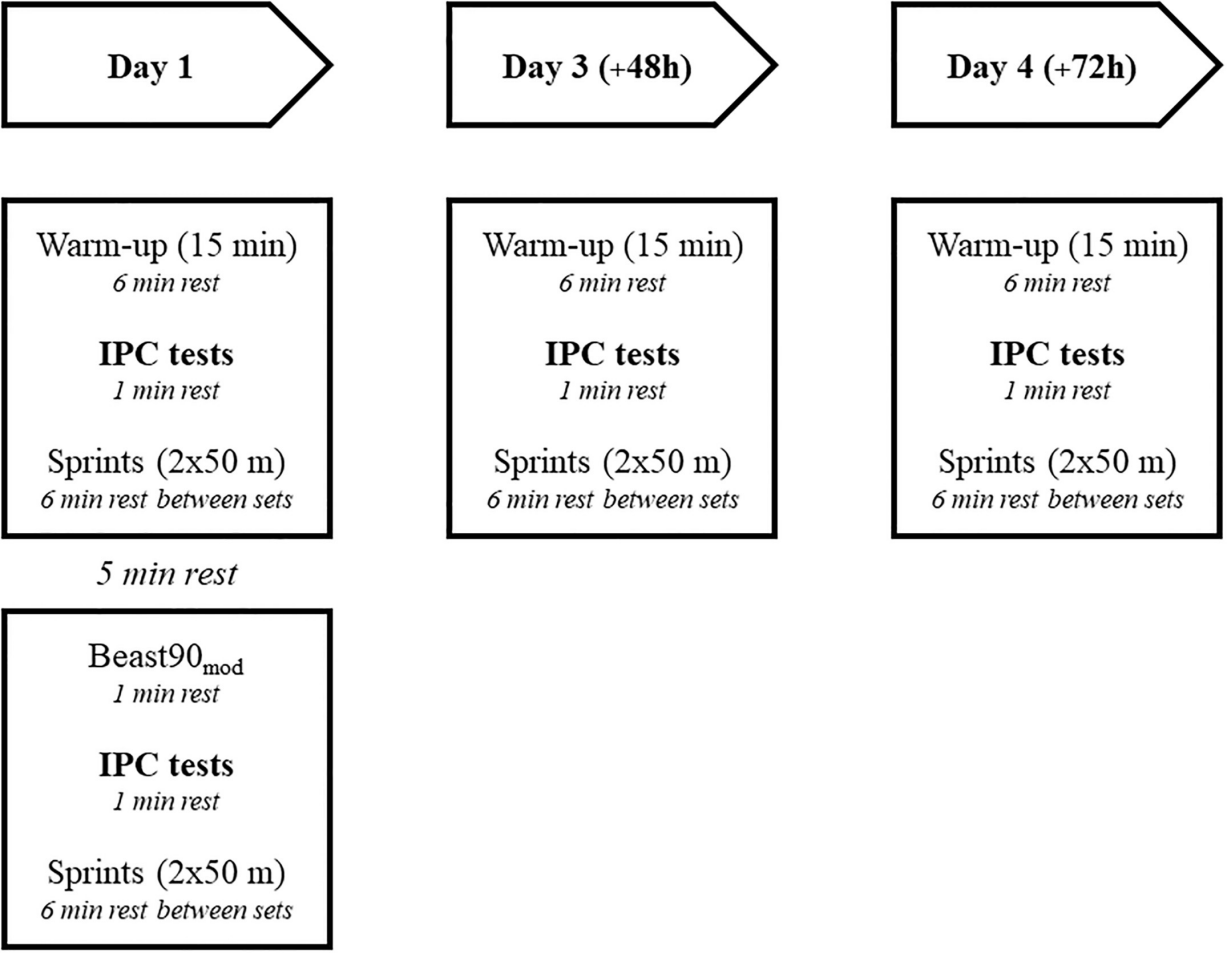
The sequence of activities. The 90:90 and 90:20 tests.

All participants were fully informed about the purpose of the study and the benefits and risks associated with the investigation and signed a written consent form. The participants in this manuscript have given written informed consent (as outlined in PLOS consent form) to publish these case details. In addition, the individuals pictured in Figs 1 and 2 have provided written informed consent (as outlined in PLOS consent form) to publish their image alongside the manuscript. Approval for the study was given by the Human Subjects Ethics Committee of the Catholic University of Murcia, Spain, in accordance with the Declaration of Helsinki (2008).

### Testing procedures

We first assessed the inter-session reliability of two 90° hip flexion: 20° knee flexion, (90:20) test protocols, performed on two different occasions, separated by 7 days. The protocols were performed after a standardized warm-up of approximately 15 mins, consisting of: 5 min of low-speed running (~10 km·h-1) followed by 3 min of lower limb muscle stretching (active and dynamic), 5 min of sprint drills and 3 progressive 6-s sprints separated by 2 min of passive rest. Subjects were randomly assigned to an order of testing and time of day that was maintained on both occasions, as was the footwear worn. The same investigators supervised both testing sessions.

### Reliability of two 90:20 protocols

For both of the 90:20 isometric posterior chain (IPC) strength test protocols evaluated, players stood with their buttocks, upper back, head and non-tested leg and heel against a wall (Fig 1A and 1B). The heel of the leg to be tested was placed on a force platform (Kistler 9286BA, Kistler Group, Winterhur, Switzerland), with the ankle in a neutral position. The non-tested standing leg stayed in contact with the wall with assistance from the tester, who applied the necessary amount of force to ensure that the player’s knee did not flex. A second researcher watched the test being performed and was responsible for confirming whether the player maintained the previously described position during the test. If for example rotation of the lumbo-pelvic region in the transversal plane was observed or the subject lost contact with the wall, the trial was considered invalid, and the test repeated.

The height of the box on which the force platform was placed, and its distance to the wall, were individually adjusted to ensure that each player was tested in a 90° of hip flexion and 20° of knee flexion position—measured using a goniometer. The two 90:20 protocols differed only in the arm position adopted during the test. In the hands-on-chest protocol (HC), subjects crossed their forearms on their chest, hands touching the opposite acromium (Fig 1A). In the hands-on-wall (HW) condition, subjects placed and maintained their palms flat against the wall (Fig 1B).

The tester gave all participants the same instructions; “to exert maximal force vertically into the force plate” and continuous and standardized verbal motivation “to push as hard as possible”for 3.5 s. Each limb was tested twice in each condition for a total of 4 trials per leg. The assessment of both legs was separated by 30 seconds while the time separating two trials of the same leg was 60 seconds. The force plate sampling frequency was set at 500 Hz with no filter/smoothing applied. Raw data was exported to a customized spreadsheet and the highest isometric force (IPF) produced in each limb and condition was extracted from each of the four trials (two trials HC and two HW for the right and left legs). The average of the IPF of each limb in each condition were used in analysis. Leg dominance was defined as preferred kicking leg.

In the second part of the study we compared the fatigue-recovery profile of the HC position 90:20 protocol and the 90°:90° supine bridge protocol following a simulated football competition. This was implemented during the first two weeks of post-season and used a within-subject, repeated measures design to investigate the effect of a simulated soccer competition, the BEAST90_mod_ [20], on IPF in the 90:20 and 90:90 tests immediately post, 48h, and 72h post. Prior to the start of the study, participants completed two familiarization sessions during in which they performed 2 sets of both the 90:20 in the HC position, (selected on the basis of its slightly superior reliability), and the 90:90 supine posterior chain test [15], followed by the BEAST90_mod,_ at walking speed, and then at regular speed. These two familiarization sessions took place during week 1, while the actual testing sequence was completed during week 2 of post-season.

#### Testing Procedures

Players completed both IPC tests on four separate occasions. 1) prior to beginning the BEAST90_mod_ (Pre); considered as the baseline, non-fatigued condition, 2) immediately following the BEAST90_mod_ (Post), representing acute fatigue 3) 48 hours post- BEAST90_mod_ (+48) and 4) 72 hours post- BEAST90_mod_ (+72), to evaluate residual match induced fatigue. As the day following competition is typically a rest or recovery day in pro soccer and would not typically incorporate neuromuscular performance tests such as these, we elected to evaluate “residual” fatigue assessment on the second day post-match (+ 48) to align with the time point at which these assessments are typically implemented. The sequence of activities is shown in Fig 2.

**Fig 3:**
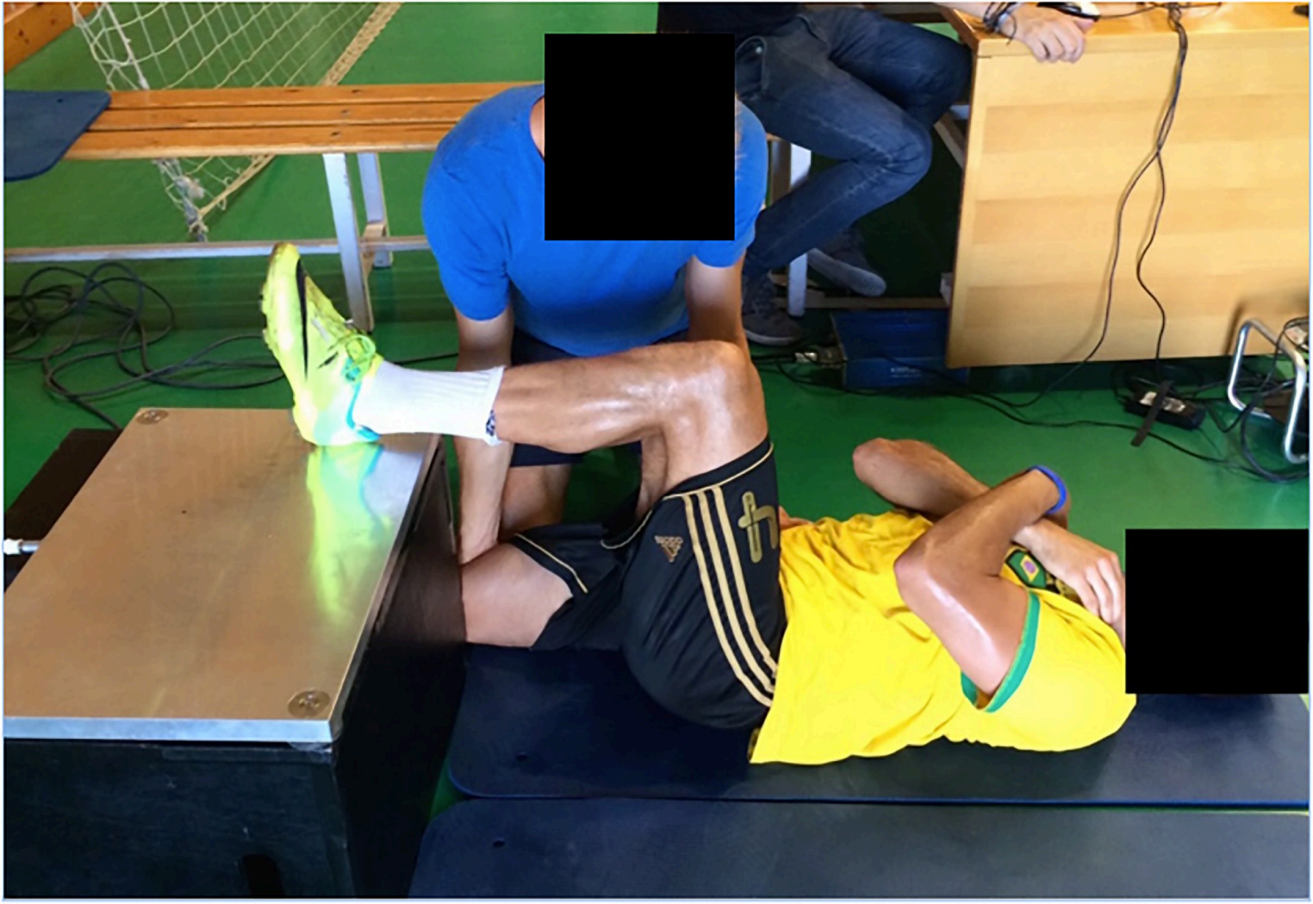
Supine 90°:90° posterior chain test [15]. Modified Ball-Sport Endurance and Sprint Test (BEAST90_mod_).

The 90:90 test (Fig 3) is performed lying on the floor in a supine position with buttocks, upper back and head in contact with floor. The heel of the tested leg was placed on the force platform with ankle in a neutral position and the thigh and hip positioned to create 90° of hip flexion and 90° of knee flexion, using a goniometer. The tester exerted pressure on the subject’s pelvis on the relaxed limb to ensure that they did not raise their buttocks off the floor. The subject and testing order was randomly determined at pre- and then maintained on all testing days. Two 90°:90° and two 90:20 trials were performed on each limb with 30 seconds of rest between trials of different legs and 60 seconds between repetitions of the same leg. Participants were given standardized instructions and verbal encouragement to encourage maximal effort for 3.5 seconds. The data was analyzed as described above so that the average of the IPF of each pair of trials were included in the analysis.

Modified Ball-Sport Endurance and Sprint Test (BEAST90mod).

We used the modified 90-minute Ball-Sport Endurance and Sprint Test (BEAST90_mod_) [20], a simulated soccer match protocol to provide a controlled and standardized workload, in contrast to a real match, during which there is a large inter-player variability in the activity profile [21]. The BEAST90_mod_ is a simplified version of the BEAST90, consisting of 2 x 45 min halves with a 15 min break between halves and reflecting the activity profile and physical demands of a regular competitive match, including periods of walking, resting, sprinting, deceleration, acceleration, backwards running, slalom running and change of direction [20].

Players were allowed to drink water during the test, at the 15 seconds resting points and at half time. A maximum of 6 players participated at each course with a minimum interval of 5 minutes between the start of each participant. Players were encouraged to maintain their maximal performance during the maximal effort sections of the protocol such as the sprints. The total number of laps completed during each half were used to calculate the total distance covered by multiplying the lap count with approximate lap distance of 171.4 meters plus the distance covered during the last incomplete lap.

#### Statistical analysis

Statistical analysis was conducted using IBM SPSS Statistics 20 for MAC (IBM Corporation, Armonk, NY, USA).

Differences between the two trials for the inter-session reliability and for differences between the groups (HC vs HW and dominant vs. non-dominant) were tested using Student’s paired t-test. The magnitude of differences between consecutive trials was also expressed as standardized mean difference (Cohen effect sizes, ES) [22]. Threshold values for Cohen ES statistics were: 0.0–0.2 = Trivial; >0.2 = small; >0.6 = moderate, and >1.2 = large. The average of both trials for each session (M1 and M2) was calculated to determine the coefficient of variation (CV) and the respective 95% confidence interval, according to the following equation:

$$\text{CV}(\text{participant},\;\text{leg}\;\text{position})=100\times \sqrt{2}\times (\frac{\text{M}1-\text{M}2}{\text{M}1+\text{M}2})$$

Fatigue-recovery IPF data are presented as mean ± standard deviations (mean ± SD). Normality was assessed with the Kolmogorov-Smirnov test and comparisons between average IPF values across time points were analyzed using General Linear Models (GLM) of repeated measures. A Bonferroni post hoc correction was then applied if a significant F-value was found. Lastly, we examined correlations between acute fatigue (Δ pre vs post) and fatigue at +48h (Δ pre v +48h) in the dominant and non-dominant and between the magnitude of force loss in the dominant compared to the non-dominant leg at these timepoints using a Pearson test. Statistical significance was set at P < 0.05.

## Results

### Reliability of HC and HW 90:20 protocols

**Fig 4:**
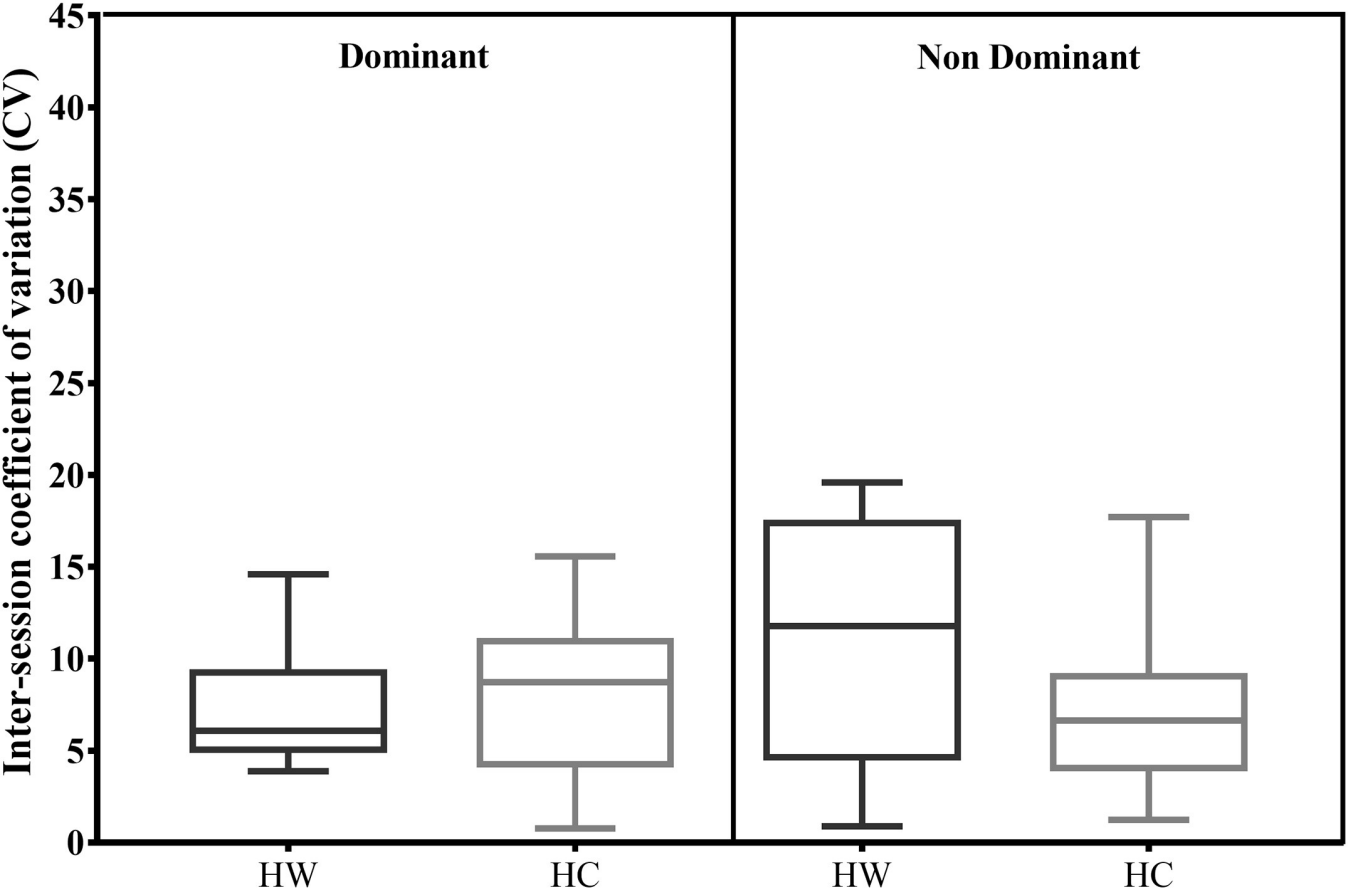
Inter-session median CV distributions with boxplots for D v ND limb in 90:20 hands-on-chest vs. hands-on-wall.

There was no significant inter-session difference in mean IPF in the dominant leg (D) for HW (Day 1: 215.8 ± 45.1 N; Day 2: 218.9 ± 51.9 N) or HC (Day 1: 232.9 ± 54.1; Day 2: 217.5 ± 50.7 N) or for non-dominant leg (ND) HW (Day 1: 220.6 ± 48.3; Day 2: 229.6 ± 60.3 N) or HC (Day 1: 232.9 ± 54.1; Day 2: 217.5 ± 50.7 N). There was also no significant difference in IPF in HW vs. HC in same leg. Inter-session CV’s were not significantly different between positions and legs (Fig 4), with differences close to 0%, indicating that the protocols have a similar variability.

Table 1 shows the inter-session coefficient of variation and 95% confidence intervals for the two 90:20 protocols. All indices of inter-session reliability showed “trivial” effect sizes.

**Table 1:** Coefficient of variation (CV), confidence interval (CI) and effect sizes (ES) for day 1 vs day 2 in D and ND legs for 90:20 HW and HC positions.

	CV	95% CI	*ES*
**Dominant leg HW**	7.3	5.46–9.14	0.06
**Dominant leg HC**	8.1	5.26–10.94	0.26
**Non-Dominant leg HW**	11.0	7.43–14.57	0.17
**Non-Dominant leg HC**	7.3	4.73–9.87	0.28

CV = coefficient of variation; CI = confidence interval; ES = Effect size; HW = Hands on wall; HC = Hands on chest

#### Fatigue-recovery profile of 90:20 v 90°:90° IPF following simulated soccer competition (BEAST90_mod_)

The mean total distance covered during the BEAST90_mod_ protocol was 8378 ± 1091, of which 586.6 ± 76.4 m was sprinting, 346.7 ± 49.8 m backward running and 312.8 ± 40.7 m slalom running.

Mean IPF at each time point for both tests and legs are shown in Table 2. In both legs in both tests there was a significant decrease in IPF post compared to pre-BEAST. At +48, 90°:20° D-leg and ND leg IPF remained significantly lower (*p* < 0.01; p ≤ 0.05) than pre-BEAST. At +48 90°:90° IPF was not significantly different from pre-BEAST in either leg in either test. At +72 IPF was not significantly different from pre-match in either test. The effect sizes displayed similar values of magnitude of small to moderate (post), small (+48h) and trivial (+72h) for both positions.

**Table 2:** Isometric peak force in 90:20 and 90°:90° tests (mean ± standard deviation) immediately post, +48 and +72 post-match.

	Pre-match	Post-match	*ES*	+48h	*ES*	+72h	*ES*
**90°:90°–Dominant**	278 ± 62	235 ± 58**	*0*.*65*	250 ± 60	*0*.*43*	270 ± 37	*0*.*12*
**90°:90°–Non-Dominant**	264 ± 65	228 ± 61***	*0*.*52*	240 ± 66	*0*.*35*	270 ± 44	*-0*.*09*
**90:20 –Dominant**	254 ± 71	203 ± 62***	*0*.*68*	221 ± 68**	*0*.*44*	258 ± 52	*-0*.*05*
**90:20 –Non-Dominant**	241 ± 71	192 ± 65***	*0*.*65*	216 ± 70*	*0*.*33*	250 ± 57	*-0*.*12*

***Significantly different from pre values (*p* < 0.001).;

**Significantly different from pre values (*p* < 0.01).

*Significantly different from pre values (p ≤ 0.05). Effect sizes (ES) were calculated comparing each time point (post, +48h, +72h) with pre-test values.

**Fig 5:**
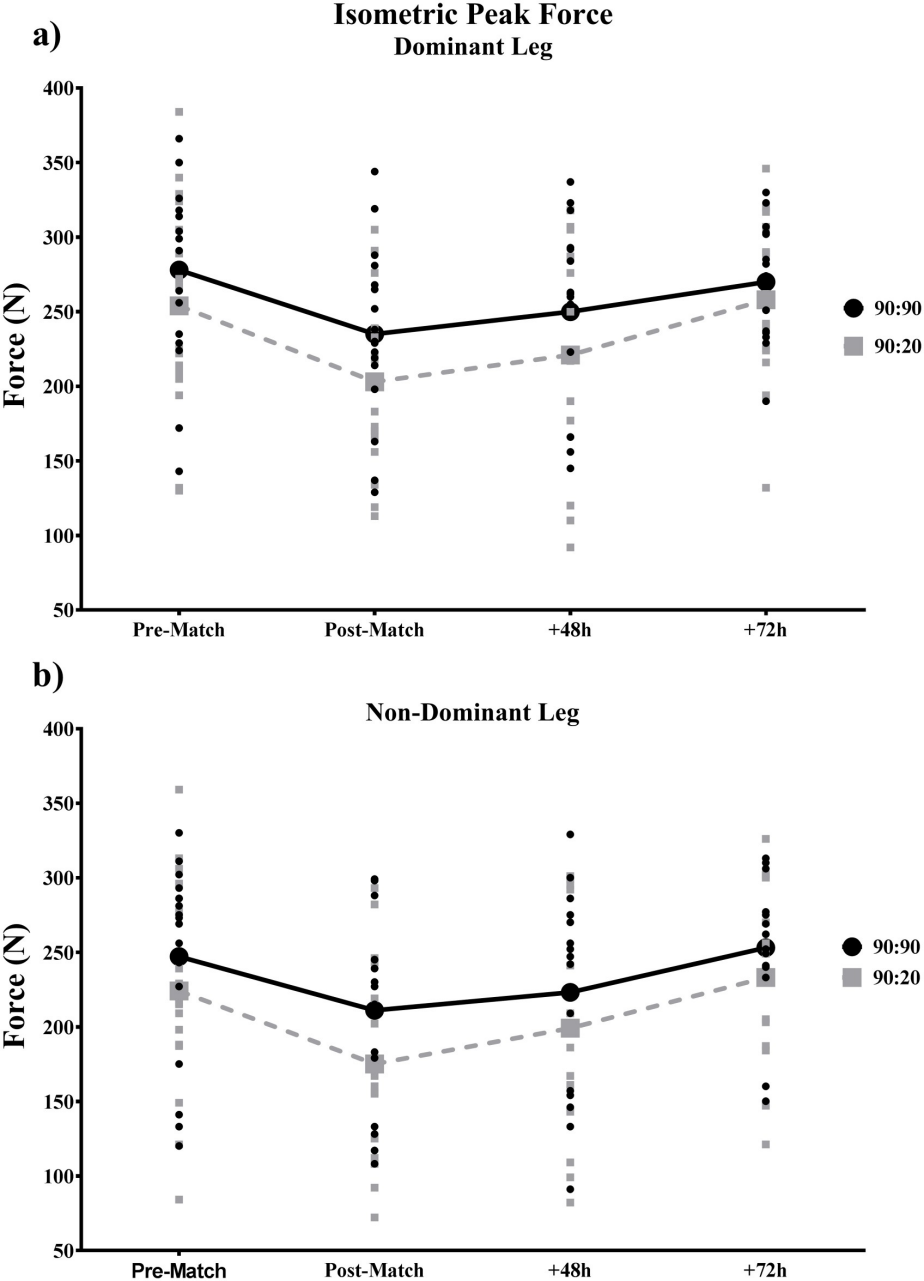
Isometric posterior chain peak force pre, immediately post, +48h and + 72h post a simulated soccer match (BEAST90_mod_) assessed with the supine 90:90 and the standing 90:20 tests (a) in the dominant leg (b) in the non-dominant leg. Thick and dotted lines represent mean values. Single data points represent individual scores for each player.

The fatigue-recovery time courses of IPF for both tests are presented in Fig 5, expressed as mean values and individual scores for each athlete in both the dominant and non-dominant leg.

### Correlations between dominant and dominant leg fatigue

There were significant correlations between the magnitude of dominant and non-dominant leg fatigue at 48h (90:90; r = 0.83, p<0.00, 90:20 r = 0.63, p = 0.01) but no correlation in acute (90:90; r = 0.40, p = 0.11, 90:20; r = 0.48, p = 0.053).

## Discussion

The main aim of this study was to compare the fatigue-recovery profile of peak force (IPF) in a novel, standing, “90°_Hip_:20°_Knee_” posterior chain isometric strength test with that of a 90°_Hip_:90°_Knee_ supine position test [15] in response to a simulated soccer match protocol, the BEAST90_mod_. We first determined the reliability of two 90:20 protocols which differed only in arm-hand placement and found that while reliability was moderate to high in both, crossing the hands across the chest (HC)–was slightly superior than placing the hands against wall (HW), and therefore used the HC protocol in the comparison of fatigue-recovery profiles of 90:20 vs 90:90. We found significant declines in D- and ND-limb IPF in both tests immediately post the simulated match, although decrements were slightly larger in the 90:20 (19.8–21.1%) than the 90:90 (13.1–15.3%). At +48 post match, IPF remained below baseline pre-BEAST values in both tests, but this was only significant in the 90:20. At +72 hours IPF in both tests had returned to baseline.

### Reliability of 90:20 procotols

The 90:20 showed moderate to high overall reliability (CV’s) in both positions but reliability was higher in the ND in both the HC and the HW, compared to the D-leg HC; and HW. Two of the participants showing particularly high inter-session variability. 90:20 HC inter-session CV’s are similar to that reported by Wollin et al. [16] in an isometric test using an externally fixed dynamometer in a prone 45:30 position, while McCall et al [15] reported substantially higher reliability in the force platform assessed supine 90°:90° position in the dominant (CV = 4.3%) and non-dominant leg (CV = 5.4%). Potentially, the more functional and less stable position of testing in the standing 90:20 may require a longer period of familiarization to improve reliability; in terms of controlling posture and learning the direction of force application compared to the trunk and hip supported bridge position. The inter-session CV distribution of both positions was however below or near 10%, the criterion generally used to qualify a variable as reliable [23]. Further research could evaluate the reliability of a supine position IPC test which uses a higher box than for the 90°:90°, allowing an increase in knee extension while also maintaining the stability that the floor provides.

### Fatigue-recovery profile of 90:20 and 90:90

The acute reductions in 90:90 IPF following the BEAST90_mod_ were of very similar magnitude to that reported by McCall et al. [15], (16% in D- and 13% in ND-legs, respectively) immediately post a 90-minute competitive match. They also reported a similar magnitude of acute fatigue in another supine IPF test which places the hamstring under greater extension at the knee (30° from full knee flexion) than the 90°:90°, but in a lower degree of hip flexion. However, Nedelec et al. [10] noted a larger effect size for force loss in the 30° position than the 90:90 at 24, 28 and 72 hours in professional players post competition. In semi-pro players who performed a 90-minute simulated match, Cohen et al. [8] observed that the largest magnitude loss of eccentric torque during seated isokinetic knee flexion at 10° of flexion—the most extended position evaluated and a similar hip and knee angle as the 90:20. However, the standing 90:20 involves active hip extension as well as active knee flexion and is proposed as a means to evaluate the combined force output of the hamstrings and by definition, other musculature involved in hip extension. Taken together with the present data, showing a larger (but not significant) acute IPF loss in the 90:20 than the 90:90, it appears that repeated sprint activity and match-play may have a larger acute impact on hamstrings/posterior chain force production when tested in a position of greater knee extension. The overall fatigue-recovery profile of IPF in the 90:20 and 90:90 was similar between baseline at 48 hours, demonstrating the sensitivity of isometric testing to acute and residual fatigue associated with high intensity dynamic activity [10,14–17]. However, at +48h, 90:20 IPF remained significantly below baseline in both D (P < 0.01, -13,0%) and ND limbs (P < 0.05, -10,4%), while deficits were slightly smaller and non-significant for the 90:90 (D: -10,1%; ND: -9,1%), respectively. Impaired force production is considered to be the best indirect marker of muscle damage including myofibrillar disruption and inflammation [24], and IPF loss following repeated eccentric contractions is strongly correlated with the proportion of fibres showing ultrastructural disruption [25]. The higher peak force of the two joint BF when in a longer length position [26], and the observation that a greater magnitude of eccentric flexion torque loss occurs in the most extended position tested [8] may explain why there are larger IPF changes in the 90:20 test, which also may better reflect the degree of muscle damage 48 hours post-match.

Regarding the timeline of recovery, our findings contrast with those of Nedelec et al. [10], who still observed significant reductions in pressure cuff assessed post- match peak, in the dominant leg in the supine 90:90 at 72 hours as well as at 48 hours post. In the present study, the recovery of IPF was evident in both tests at 72 hours after the simulated match protocol, with effect sizes displaying trivial values of magnitude (ES: -0.09–0.2) (Table 2). The discrepancy with Nedelec’s findings could be explained by the lower total (8378 m v 10–13000 m) and sprint distance (586.6 m v 650 m) in the BEAST_mod90_ compared to a match [20,21] and as such our results may slightly underestimate the degree of fatigue to hamstrings/posterior chain experienced by players in a real competitive match. On the other hand, a recent review concluded that despite matches resulted in a greater magnitude of muscle damage and inflammatory and immunological response, neuromuscular performance deficits did not significantly differ from that observed after simulation protocols [27]. Indeed, Wollin et al. [16], observed a more rapid recovery of IPF at 48 hours in elite U17 players after a competitive match than in the present study, with a mean IPF deficit of < 6%, which was not significantly different from pre-match values. Moreover, Nedelec [10] found no significant correlations between posterior chain dominant leg IPF decrements and the number of sprints or changes of directions or volume of high intensity running completed. Therefore, in relation to posterior-chain neuromuscular function, further work is needed to understand the relationship between components of match load and residual fatigue, which, at least when quantified by deficits in jump performance, is associated with decreased high intensity running performance in subsequent training or competition [28,29]. It is important to note that player physical characteristics such as aerobic fitness [30] and lower body strength [31] have also been found to mediate the magnitude of jump-performance assessed post match fatigue in team sport athletes. While, interactions between player fitness or neuromuscular characteristics and fatigue-recovery kinetics in the posterior chain test have not been reported, Delextrat et al. [31] reported that increased hamstrings strength-endurance reduced the magnitude of acute eccentric hamstring fatigue following a simulated match, but it is not known if this characteristic would also mediate the magnitude of residual fatigue. Further investigation of the interaction between fatigue response to/recovery from competition, and aspects of hamstrings neuromuscular performance—including strength, rate of force development or strength-endurance, and other potential mediators such as fascicle length [32] is warranted.

### Associations between dominant and non-dominant limb fatigue recovery profiles

Consistent with previous studies [10,14–16], the magnitude of fatigue was slightly higher in the dominant versus non-dominant leg. Interestingly, there were no association between acute IPF decline in the two limbs, but significant correlations were observed at +48h. These divergent associations between dominant and non-dominant limb fatigue at the two timepoints may reflect the different mechanisms underlying acute versus residual fatigue. Neuromuscular fatigue during and following soccer competition is a consequence of some combination of peripheral fatigue—metabolic and mechanical damage that impairs the excitation-contraction coupling and the contractile process, and central fatigue—the capacity of the central nervous system to activate muscle [33]. A leg preference reported in sprint deceleration and shown to promote the development of inter-limb hamstring strength asymmetries [34] could also underlie an asymmetry in mechanical damage and in the magnitude of this component of peripheral fatigue. Potentially, the divergent IPF dominant v non-dominant fatigue-recovery relationships observed may reflect the relative contributions of these components of fatigue in each leg and the variable time course of recovery. They may also be influenced by asymmetries in physical characteristics; with a substantial asymmetry in the (acute) fatigue resistance of the dominant versus non-dominant hamstring observed in semi-professional players [35]. Inter-limb asymmetries in acute and residual fatigue warrant further investigation as they may inform conditioning and recovery strategies.

Acute, residual fatigue/inadequate recovery from competition is thought to explain the higher incidence of injury in the latter part of matches [12] and in match congested periods [13]. Easy to implement isometric tests can be integrated as part of in-season posterior chain neuromuscular performance monitoring to rapidly define normal patterns of fatigue within a team and identify individuals showing abnormal changes in total force and asymmetries. This data, in conjunction with other objective and subjective measures which help define player response to competition and training load, could inform and influence recovery strategies/modify player load within match training micro cycles to minimize impact of residual/accumulated fatigue on performance and injury risk [36,37]. By definition, post and residual decrements in muscle strength are a valid means to quantify neuromuscular fatigue, however an association between weekly changes in strength in-season and injury has only been demonstrated in a case report [14]. Schache et al. [14] reported that relative to trends in the whole team, an abnormally large change week to week increase in posterior chain IPF asymmetry in weekly in-season testing preceded an HSI. Nonetheless, while it is proposed that isometric strength, in particular during the late swing phase of the sprint cycle, has an important role in hamstrings injury risk [38], IPF cut points for risk have not been determined as they have for the Nordic hamstring exercise [5] or isokinetic testing [7], and as such these tests are not currently advocated as a risk stratification tool in primary prevention.

It has been suggested that eccentric hamstring strength should be evaluated and developed specifically at longer muscle lengths, to protect from overstretch mechanism hamstring strain injuries [39,40]. Furthermore, Brockett et al. [41] noted deficits (relative to the uninjured limb) in long-length isokinetic hamstring torque, but not in peak torque in athletes with recurrent hamstring strains. Long length IPF assessment may have a role in secondary prevention, to track progress during rehabilitation and support return to play decisions following hamstring injury [42], and an IPF deficit (relative to healthy limb) at 15°, but not at 90°, was identified as a prospective risk factor for re-injury [43]. Isometric testing such as the 90:90 or 90:20 can be performed rapidly enough to frequent monitoring feasible in sports or clinical environments, with tests such as the 90:20 a means to specifically identify long length deficits, either in isolation, or potentially in a long length:shorter length test peak force ratio.

Some limitations of the present study should be addressed. Firstly, the small sample size (based on an opportunity sample) may have limited our ability to detect significant differences in the reliability of the two 90:20 protocols. Secondly, the absence of reliability data for the 90:90 with this study’s sample would have allowed a direct comparison between these protocols. However, given that the reliability of the 90:90 test had been previously presented in the literature, and adding a 3rd inter-session reliability protocol would have placed too high a demand on the players time, we elected to focus on the comparison of the two potential positions in the novel 90:20 protocol.

## Conclusion and practical implications

Our results support findings showing that a simple 90°:90° posterior chain force platform isometric test can detect acute and residual fatigue related to match play in footballers [10,14–17]. Based on a comparison of the peak force recovery kinetics in this 90°_hip_:90°_knee_ test with that of a novel 90°_hip_:20°_knee_ standing test, our data also suggests that match play induces a larger magnitude/more sustained disruption of force production at longer hamstrings length, in a position of greater knee extension. Testing posterior chain IPF in a more extended position may provide increased sensitivity in the assessment of match play induced fatigue, and potentially in identifying residual deficits following HSI [43]. However, the 90:20 test may require a longer period of familiarization than the 90°:90° supine bridge posterior chain test. Implementing either similar test may help to define tolerance to, and from recovery from, competition and training load in football and other team sport athletes.

## Supporting information

S1 File Reliability study raw data: Coefficient of variation calculations for the 90:20 protocol. (XLSX)

S2 File Fatigue-recovery profile raw data: Individual values for each participant on the different time points using the 90:20 and 90:90 protocols. (XLSX)
